# Surgical Planning in Penetrating Abdominal Crohn's Disease

**DOI:** 10.3389/fsurg.2022.867830

**Published:** 2022-05-03

**Authors:** Pär Myrelid, Mattias Soop, Bruce D. George

**Affiliations:** ^1^Department of Surgery, Linköping University Hospital and Department of Biomedical and Clinical Sciences, Linköping University, Linköping, Sweden; ^2^Department of Surgery, Ersta Hospital, Karolinska Institutet at Danderyd Hospital, Stockholm, Sweden; ^3^Department of Colorectal Surgery, Oxford University Hospitals NHS Foundation Trust, Oxford, United Kingdom

**Keywords:** Crohn's disease, perforating disease, surgery, complications, optimisation

## Abstract

Crohn's disease (CD) is increasing globally, and the disease location and behavior are changing toward more colonic as well as inflammatory behavior. Surgery was previously mainly performed due to ileal/ileocaecal location and stricturing behavior, why many anticipate the surgical load to decrease. There are, however, the same time data showing an increasing complexity among patients at the time of surgery with an increasing number of patients with the abdominal perforating disease, induced by the disease itself, at the time of surgery and thus a more complex surgery as well as the post-operative outcome. The other major cause of abdominal penetrating CD is secondary to surgical complications, e.g., anastomotic dehiscence or inadvertent enterotomies. To improve the care for patients with penetrating abdominal CD in general, and in the peri-operative phase in particular, the use of multidisciplinary team discussions is essential. In this study, we will try to give an overview of penetrating abdominal CD today and how this situation may be handled. Proper surgical planning will decrease the risk of surgically induced penetrating disease and improve the outcome when penetrating disease is already established. It is important to evaluate patients prior to surgery and optimize them with enteral nutrition (or parenteral if enteral nutrition is ineffective) and treat abdominal sepsis with drainage and antibiotics.

## Introduction

Crohn's disease (CD) is a transmural inflammatory bowel disease (IBD) characterized by discontinuous inflammation that may involve any part of the gastrointestinal tract (1, 2). Abdominal pain, bowel obstructions, and diarrhea are generally the dominating symptoms and there is quite often a time lag between the onset of symptoms and the eventual CD diagnosis (3). Patients with CD may also develop weight loss and malnutrition, which is due to less food intake, inflammatory activity, and sometimes by-passes created by intestinal fistulas. Fistulas, intestinal as well as perianal, are quite frequent, and after 20 years of disease duration, up to 50% of patients may have suffered at least one episode of fistulation (4–6).

The *classical* CD was described by Crohn et al. (7) as terminal ileitis, sometimes also involving the caecum. The location of CD within the gastrointestinal tract seems to have changed somewhat over time and today the Montreal classification (Table 1) is used as a way to describe the different locations as well as different forms of severity and complexity of CD (8). In relative measures, the *classical* CD of the terminal ileum and caecum has decreased in favor of colitis as well as the spread of the disease to both the small bowel and colon outside of only the caecum (L in Table 1) (10, 11). Apart from the change in the location of the disease, there are also a number of reports on a shift toward more patients diagnosed either very early or late in life (10, 12). as well as less frequent occurrence of stricturing or penetrating disease (A and B in Table 1) (10).

**Table 1:** Montreal classification of Crohn's disease (8).

	**Montreal classification of Crohn's Disease**			**Disease modifier**
Age at diagnosis (A)	A1	A2	A3	N/A
	≤ 16 years of age at diagnosis	17–40 years of age at diagnosis	>40 years of age at diagnosis	
Location of the disease (L)	L1	L2	L3	L4
	Ileal or ileocecal disease	Colonic disease	Ileocolonic disease (other than ileocecal)	Isolated upper gastrointestinal disease
Behavior of the disease (B)	B1	B2	B3	p
	Inflammatory, non-stricturing, non-penetrating disease	Stricturing disease	Penetrating disease	Perianal disease

*The “worst” type of classification ever taking part in a patient's life will be the one used in the classification, i.e., a patient diagnosed at age 12 with the inflammatory ileocaecal disease will at first get the classification A1 L1 B1. When the same patient later in life develops colonic disease as well and develops an ileo-sigmoidal fistula and also a perianal fistula the new classification will be A1 L3 B3p*.

*In pediatric Crohn's disease, the modified Paris classification is used by dividing e.g., small bowel disease to L1 if the last 1/3 of the ileum is affected and L4b if the small bowel is affected in the proximal 2/3 of ileum up to the ligament of Treitz. Upper gastrointestinal Crohn's disease proximal to the ligament of Treitz is classified as L4a (9)*.

The medical treatment of CD has in some ways gone from a gradual step-up of medication from steroids, via immunomodulators (e.g., azathioprine, mercaptopurine, and methotrexate), to biologicals (e.g., infliximab, adalimumab, golimumab, vedolizumab, and ustekinumab) (13, 14) to a more rapid step-up, or even a “top-down-approach” in selected patients (15, 16). Whether this strategical change has modified the course of the disease or the need for surgery is still debated (17–20), and at the same time, there has been a vast change in the costs for the healthcare of patients with IBD (21).

When it comes to surgical therapy of CD, the indications have mainly been the treatment of complications to the disease, e.g., stricturing and/or penetrating disease (Table 1). Prior to the wider spread of immunomodulating and biological therapy, about 50% of patients underwent abdominal surgery within the first 10 years of their CD diagnosis (22, 23). Decreasing surgical rates have been reported during the last 50 years, and in a recent study from Sweden, the cumulative incidence of abdominal surgery within 10 years of diagnosis was 21% in patients diagnosed between 2004 and 2009 and 15% had a repeat abdominal procedure within 5 years from the primary operation (24, 25). Ileocaecal resection is still the most common procedure in CD accounting for 66% of the primary procedures (25).

A recent randomized controlled trial performed in the Netherlands found laparoscopic ileocaecal resection to be equally good as medical therapy with infliximab infusions regarding the quality of life, hospital admissions, and complications in patients with non-stricturing terminal ileitis who had failed immunomodulating therapy (26). In most cases, both surgery and biological therapy may be reasonable options as a first-line treatment of ileocaecal CD, and it may today rather be down to the choice of each individual if surgery or biologic therapy should be the first step after failing immunomodulation. Regardless of the chosen primary treatment modality, the patients should be informed of the probable need for the other treatment as well in the longer run. Some patients are more prone to have an early recurrence of CD after a resection, e.g., those who continue to smoke (27, 28), have abdominal (28, 29) or peri-anal (30, 31) penetrating diseases, a short duration between CD diagnosis and surgery as well as between primary and repeat surgery (30–32).

## Abdominal Penetrating Crohn'S Disease

Abdominal penetrating CD may range in complexity from an abdominal inflammatory mass (8), free perforation, entero-enteric or e.g., entero-cystic to enterocutaneous fistulas (ECF), with or without intestinal failure (IF) (33, 34).

A free perforation is uncommon in CD, in previous reports ranging around 1–2% of patients (35–38). Despite this, half of the patients developing a free perforation had this as their presenting symptom of the disease (39). The most common location is the small bowel, predominantly the ileum, proximal of a stricture but colonic perforations occur as well and might also be due to concurrent colorectal cancer (35–38). There have also been some signs of free perforations being more common in patients on biological therapy (40), but it is still unclear if this is due to the drugs themselves, long-standing inflammation (with only partial or no response to medical therapy) or merely an association between more active disease and the need for biological therapy.

**Figure 1:**
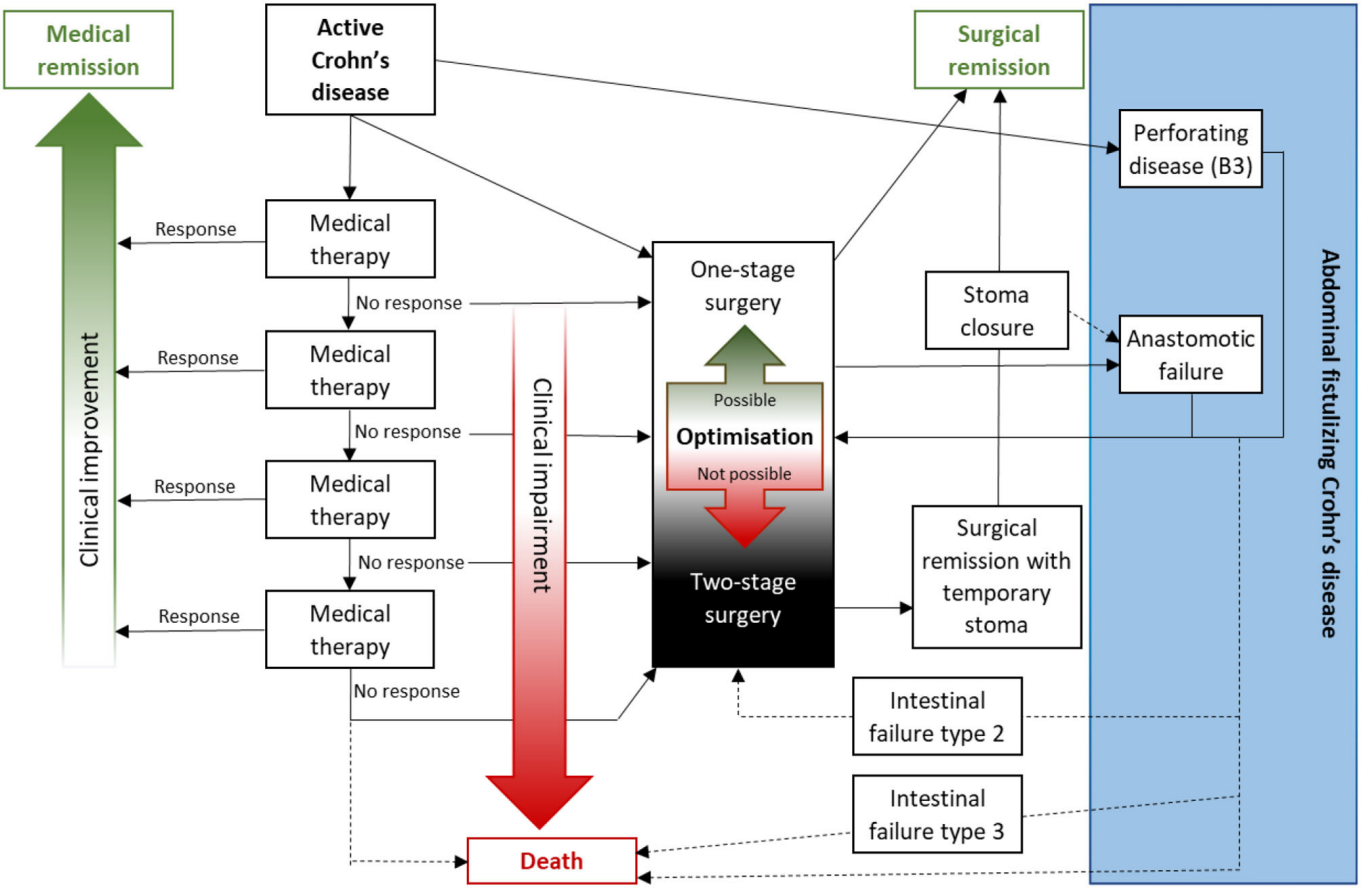
Abdominal fistulizing Crohn's disease may develop due to penetrating disease but more often due to surgical complications. With increasing non-responsive attempts with medical therapies (e.g., steroids, immunomodulators, and/or biologicals) patients may develop clinical impairment with an increasing number of surgical risk factors like weight loss, hypo-albuminemia, or penetrating disease. Before deciding on a primary anastomosis or not the risk of anastomotic dehiscence should be evaluated as well as if the patient is fit enough to survive such complication or not. The patient must be fully aware of such risks as there is a risk of severe post-operative morbidity and mortality. In a patient deemed not suitable for surgery with primary anastomosis pre-operative optimization (e.g., enteral or parenteral nutrition, drainage of collections, and antibiotics) may change this and otherwise patients should be advised toward two-stage surgery with two-barrel stoma (of the future anastomosis) or possibly a covering stoma.

The most common type of penetrating disease is the finding of an inflammatory mass seen on abdominal CT or MR enterography where it may be found in about 15–33% of patients after 10 years from their CD diagnosis (41–43). In a recent population-based database analysis of fistulizing CD, it is estimated that ~77, 000 patients in the US have a fistulizing disease with a cumulative incidence at 20 years from CD diagnosis of 50% (44). The most common type of fistulizing disease is the perianal ± vaginal component (which is not further covered in this paper), while 6 and 31% of the patients with CD have ECF or internal fistulizing disease, respectively (44). There are reports on increasing numbers of operations due to fistulizing disease after the introduction of biologicals in CD; in the US, a 60% increase was seen between 1993 and 2004 (45). Just like in the case of free perforations (40), it is unclear if this increase is drugs induced or due to long-standing inflammation or an indication bias. In a report on the effect of delay of surgery in CD Iesalnieks et al. could show that patients with pre-operative active disease for >5 months pre-operatively had a higher number of structures involved in the inflammatory mass and a higher incidence of post-operative septic complications (31 vs. 13%, *p* < 0.002) in comparison to those with a shorter duration of pre-operative active disease (46). This indicates rather that it is the non-response to medical therapy and the clinical deterioration that may be of importance regarding the development of complicated penetrating CD (Figure 1).

The other major cause of penetrating CD is due to surgical or endoscopic complications (47–51). The most advanced form of penetrating CD developing IF is related to surgical complications in 83–88% and only 12–17% due to the disease itself (33, 34). Most patients with CD and post-operative complications will, however, not develop IF but rather intra-abdominal septic complications (IASC), like abscesses and/or anastomotic fistulas, occurring in about 5–10% (47–50). Any patient with CD can develop IASC, but a number of peri-operative risk factors have been identified, e.g., penetrating disease, smoking, anemia, weight-loss, hypo-albuminemia, and colonic anastomosis (49, 52, 53). When it comes to pre-operative medical therapy, it is quite evident that steroids increase the risk of IASC as well as impair the anastomotic healing (48, 50, 54), while the evidence is less convincing regarding immunomodulators and biologicals and probably associated with indication bias (47, 55, 56). Moreover, the risk of anastomotic complications increases with the number of risk factors present at the time of surgery, approaching 50% in the event of three or four risk factors (47, 49).

It is important to evaluate the patient prior to surgery and to determine if they are deemed fit for anastomosis or not, if pre-operative optimization is possible or if a delayed stoma may be a better option (57). Some patients will, however, be less prone to accept even a temporary stoma, and in such situations, it is of important to evaluate the risk from case to case regarding IASC. There are also indications that a split stoma is a safer method than anastomosis with a proximal diverting loop-ileostomy when it comes to post-operative complications (58). Some patients may be fit to tolerate a major complication and morbidity, sometimes including repeat laparotomy/laparotomies and possibly intensive care, why a calculated risk may be justified. Others will, due to, e.g., impaired general condition or old age, not have enough reserves to manage severe complications which there will be a risk of mortality in case of an IASC. All patients, but especially high-risk patients, deserve to be discussed in a multidisciplinary team conference (MDT) prior to surgery (59–61). Prior to the surgical decision on anastomosis or not there should be informed consent of the patient on the surgical and perioperative plan (61, 62) and one must always remember that calculated risks must be the decision of the patient, as he/she is the one who will suffer the possible consequences.

## Pre-operative Optimization Of The Patient

### Contemporary Outcomes in Surgery for Crohn's Disease

Contemporary observational data suggest that outcomes from intestinal surgery for CDcan be improved. In 2017, the European Society of Coloproctology reported a detailed snapshot study of 315 people undergoing elective or emergency ileocaecal resection for CD in 151 centers (63). Among the striking findings were 30-day incidence rates of post-operative abdominal sepsis of 9% and reoperations of 6%. Similarly, a French multi-center study demonstrated a 30-day incidence of abdominal sepsis of 18% and a reoperation rate of 7% (64). A German study on colorectal surgery, other than ileocaecal resection, reported an abdominal sepsis rate of 11% and a reoperation rate of 21% (65).

Most colorectal surgeons mainly manage colorectal cancer. In that clinical context, abdominal sepsis rates of 9–18% and reoperation rates of 6–21% seem unacceptable. Indeed, an analysis of data from the American College of Surgeons National Surgical Quality Improvement Program, a prospectively collected clinical registry, directly compared outcomes in intestinal resection for CD and ulcerative colitis with outcomes in colorectal cancer surgery (66). Although patients operated on for IBD were younger (median of 40 vs. 66 yrs), and less co-morbid (American Society of Anesthesiology score of I-II of 59 vs. 42%), they had inferior post-operative outcomes. The median post-operative length of hospital stay was 5 vs. 4 days and, more significantly, 13 vs. 9% were readmitted and 6 vs. 3% reoperated within 30 days, differences which remained on multivariable analysis.

Intestinal failure, necessitating long-term parenteral nutrition, is a real risk in CD. CD remains the most common diagnosis leading to chronic intestinal failure (67), and post-operative abdominal sepsis is the most common mechanism of this life-altering complication arising (34).

Studies of current practice thus demonstrate that there is a quality issue in surgery for contemporary CD. The European Society of Coloproctology study discussed above provides further data that help explain why this is the case (63). About 18% of patients had an abdominal abscess, but only 5% were drained before surgery. About 18% received high-dose corticosteroids at the time of surgery. In 8.5%, parenteral nutrition was administered prior to surgery, signaling significant undernutrition. Despite such risk factors in a substantial portion of patients, 89% had a primary anastomosis. Pre-operative abscess, corticosteroid therapy, and undernutrition are all among a range of risk factors that have been identified as increasing the risk of suffering septic and major complications in CD surgery.

Many of those risk factors are modifiable (68). The concept of pre-operative optimization has evolved to describe clinical pathways that address a range of modifiable risk factors prior to surgery. Due to the nature of the risk factors in CD surgery, such optimization often takes 3–6 weeks. Below, important interventions in such pathways will be discussed.

### Managing Pre-operative Abdominopelvic Abscesses and Phlegmons

**Figure 2:**
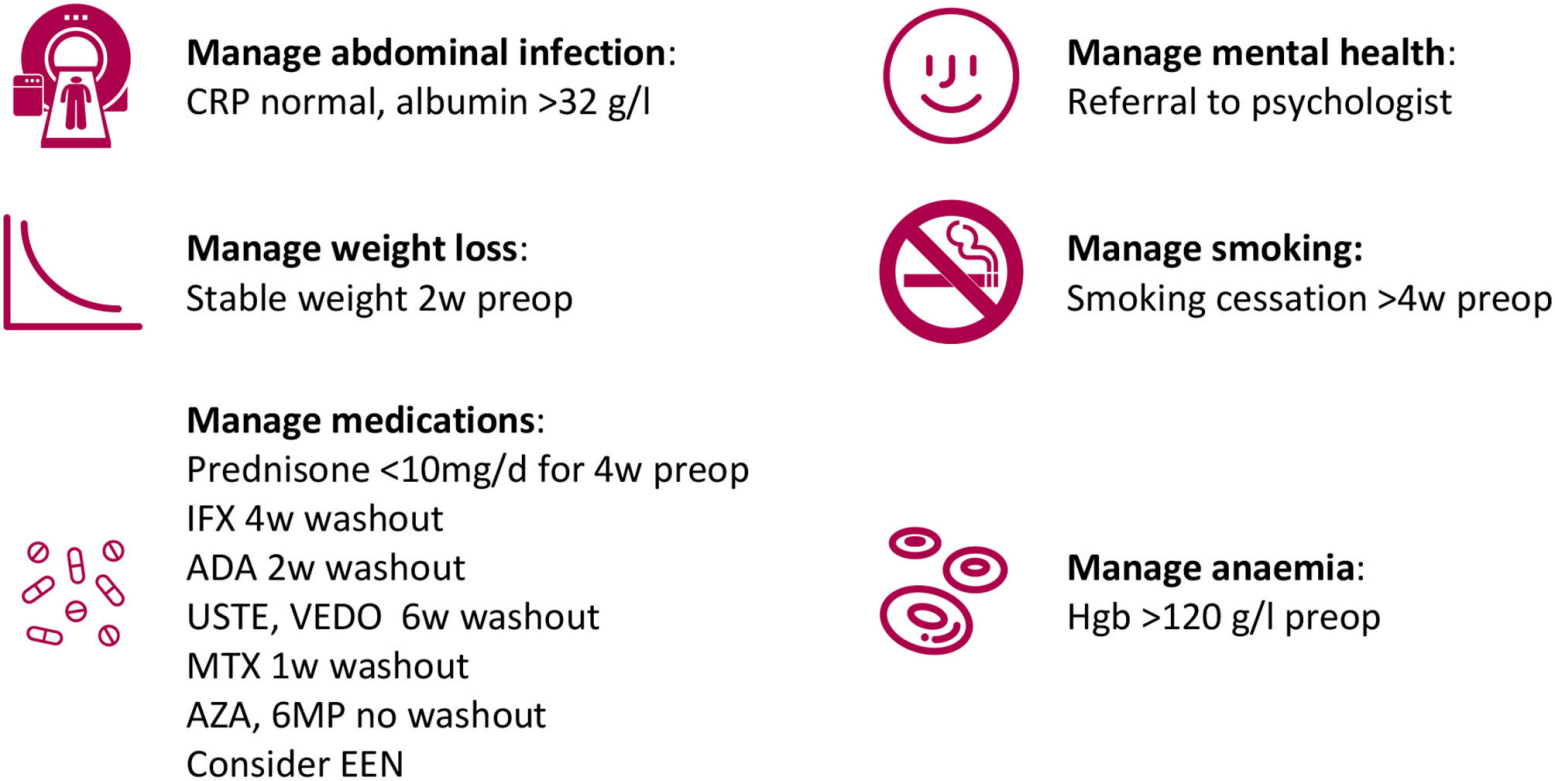
Example of pre-operative optimization components.

While a palpable abdominal mass in a person with CD was previously considered an indication for surgery without delay, today the contrary is true (Figure 2). Operating on a CD complicated by an abscess or a phlegmon or without pre-operative downstaging is technically challenging and likely to require a laparotomy rather than a minimally invasive approach. Furthermore, it is likely to require resection of healthy loops of bowel adherent to the inflammatory mass. Sometimes even a temporary diverting ileostomy, prior to a later planned resection, can improve the situation by settling the abdominal sepsis as well as decreasing the amount of inflamed bowel and possibly limiting the intestinal resection (69, 70).

Most importantly, the presence of pus in the operative field at the time of anastomosis has been shown to increase the risk of anastomotic dehiscence following small and large bowel resection for CD in several studies (48–50).

### Interventional Radiology

Percutaneous drainage guided by ultrasound or computed tomography is the main treatment modality for abscesses of significant size. The minimum size that is considered appropriate for percutaneous drainage varies depending on clinical setting and clinician preferences. It has been suggested that abscesses larger than 3 cm should generally be drained (71). A pooled series of post-operative abscesses indicates that percutaneous drainage in the abdomen is nearly always possible: it was successful in 87% of patients selected for this intervention (72), although 20% required more than one drainage insertion. It is worthwhile to remember that novel approaches are developing to reach less accessible sites, such as the transgluteal and transrectal routes (73, 74).

### Restricting Oral Diet

Although evidence is lacking on this measure, it is prudent to restrict oral diet in penetrating diseases complicated by abscesses or phlegmons. The options for meeting nutritional demands are enteral and parenteral nutrition. In the distal small bowel, residue-free enteral nutrition is preferred. The oral route is used unless compliance is poor, in which case enteral tube feeding can be considered.

When the duodenum or proximal jejunum are involved, the authors use complete bowel rest with total parenteral nutrition. Gastrointestinal secretions are further reduced by high-dose proton pump inhibitors and, particularly in duodenal defects, somatostatin analogs.

### Antimicrobial Therapy

The above approaches should be combined with antimicrobial therapy. In contrast to post-operative abscesses and abscesses of other etiology, where the duration of antimicrobial therapy should be brief after source control (75), therapy in this setting of pre-operative treatment of penetrating CD should likely be extended, in particular when drainage is not feasible, e.g., a retro-peritoneal mass rather than an abscess.

When it comes to pre-operative antibiotic prophylaxis, most guidelines on colorectal surgery, in general, propose the use of intravenous antibiotics alone (76). There are, however, some limited evidence from a recent randomized controlled trial in CD surgery suggesting a favorable outcome with the combination of mechanical bowel preparation together with both peroral and intravenous antibiotics (77). A decreased risk of incisional surgical site infections was found while no difference was seen regarding intra-abdominal infections, and further, only patients going through open surgery were included in the study. When it comes to the use of bowel preparation guidelines alone guidelines advise against the routine use in colonic surgery, but if it is to be used there is some evidence that it should be combined with oral antibiotics (76).

## Managing Disease-Modifying Medications

### Corticosteroids

Although corticosteroids should not be used to maintain remission in CD, a recent study performed in the UK revealed that one in four patients receive extensive courses of corticosteroid therapy (78). Such steroid dependency is a common indication for surgery.

Among the medications used to treat CD, corticosteroids are the class that is strongest linked to perioperative complications. Adjusting for disease severity and other confounders, such as malnutrition, is essential when evaluating links between any perioperative medication and post-operative outcomes, requiring univariable analysis and relatively large studies with meaningful event rates.

Yamamoto et al. first demonstrated the link between corticosteroid therapy and post-operative abdominal sepsis in 2000 (49). Corticosteroid therapy was defined as any dose for a month or more prior to surgery. The rates of post-operative abdominal sepsis were 18 vs. 11% in patients with and without pre-operative corticosteroid therapy (univariable *p* 0.02), and this difference remained significant on multivariable regression including markers of disease severity (37). Similarly, a study from Oxford, UK, showed that 4 weeks or more of corticosteroid usage, defined as at least 10 mg of prednisolone or equivalent per day, was an independent risk factor for anastomotic-associated complications (OR 2.67) (50). Several additional studies have shown similar results (48, 64, 79).

The Oxford study provides a pragmatic approach to pre-operative corticosteroid therapy; it is prudent to aim to reduce the dosage to 10 mg of prednisolone, or equivalent, during the four weeks preceding surgery in order to allow safe primary anastomosis (Figure 2).

### Anti-tumor Necrosis Factor Agents

The impact of pre-operative anti-TNF agents on post-operative outcomes, including abdominal sepsis, is less clear than that of corticosteroid therapy. Again, adjusting for disease severity is essential (56). Another challenge in reading the available literature is that studies typically define “preoperative” use as a dose given within 12 weeks before surgery. As anti-TNF agents have a half-life of 1–2 weeks, this generous definition may dilute any effects. As a result, recent meta-analyses of published literature suffer from a significant heterogeneity (I2 23–64%) and are contradictory in their conclusions (80). Three recent reviews conclude that pre-operative anti-TNF therapy is associated with an increased rate of post-operative infectious complications (81–83), while one did not find such an association (84). National and international guidelines are also contradictory regarding the management of pre-operative anti-TNF therapy (55, 85).

Given the grave consequences of post-operative abdominal sepsis, and the inconclusive data at this point, a pragmatic approach is to schedule intestinal surgery requiring anastomosis at ~0.5–1 dose interval after the most recent administration and delay the next dose by 2 weeks.

### Immunomodulators

Immunomodulators encompass the thiopurines azathioprine and 6-mercaptopurine, and methotrexate. The literature is relatively conclusive that thiopurines pose no additional risk when used pre-operatively (86). Methotrexate is more potent, and many authors withhold therapy for a week prior to surgery although data to guide management is scarce (47, 87).

### Exclusive Enteral Nutrition

Exclusive enteral nutrition (EEN) is a term describing nutrition delivered exclusively through liquid feeds, typically polymeric sip feeds. Although sometimes thought of as a nutritional intervention, EEN is used in IBD primarily as an immunological therapy. It is routinely used as first-line treatment to induce remission in CD in children, with an efficacy approximately that of steroids in this age group (88). In adults, EEN is not routinely used due to inferior efficacy, likely due to decreased compliance.

However, in the limited period leading up to an operation, EEN has advantages in selected adults with CD. It provides nutrition and can be relatively finely tuned to offset any ongoing weight loss. Importantly, EEN can prevent disease flares and perhaps reduce inflammatory burden in patients during the weeks when steroids are weaned and anti-TNF therapy is delayed. The limited data available so far suggests that this is the case. The studies are observational only, sometimes with historical controls.

The first study evaluating pre-operative EEN in CD was published in Nanjing, China, as recently as in 2014 (89). In this study by Li et al., all patients underwent resection of an enterocutaneous fistula caused by CD; 55 received EEN through naso-gastric tube 3 months before surgery, and 68 did not. In this severely ill cohort, EEN was associated with marked improvements in serum albumin and c-reactive protein (CRP) concentrations compared to the control group. Post-operative intraabdominal septic complications occurred in 3.6 vs. 17.6% (*p* = 0.02) (89). The Nanjing group then published similar findings in more routine intestinal surgery for CD (90).

A 6-week course of EEN was used in a study from Exeter, UK, including stricturing and penetrating disease and comparing to matched historical controls (91). Treatment was tolerated by 94%. Interestingly, 13 of 51 (25%) of patients in the EEN group improved to such a degree that they avoided surgery altogether, and were re-started on a normal oral diet. When the 38 patients who underwent surgery were compared with 76 control who proceeded to surgery shortly after listing, this study too demonstrated significant advantages in terms of pre-operative CRP concentrations. Operating time was shorter in the EEN group (3 vs. 3.5 h, *p* < 0.001), suggesting more favorable conditions in the operative field. This group also experienced fewer anastomotic leaks (3 vs. 20%, *p* = 0.019), a large difference that remained on multivariable analysis (91).

The authors routinely use exclusive enteral nutrition in two specific situations: first, to minimize inflammation in patients weaning from corticosteroids and/or biologic therapy; secondly, as part of the treatment of ileal penetrating disease. Standard polymeric sip feeds are prescribed to meet, or slightly exceed caloric and protein requirements. Only clear fluids are allowed in addition to the sip feeds. Compliance, body weight, serum albumin, and CRP concentrations are monitored every 1–2 weeks.

## Nutritional Support

### Management of Pre-operative Weight Loss

When nutritional support is provided purely to ensure adequate intake of calories and protein, EEN is not necessarily required. Instead, nutritional support is provided as, in order of preference, oral dietary supplements complementing a normal diet, oral liquid diet, enteral tube nutrition, or parenteral nutrition (55).

Nutritional status should be screened by one of several simple tools, such as the subjective global assessment or the Malnutrition Universal Screening Tool (www.bapen.org.uk), as pre-operative weight loss is an independent risk factor for anastomotic dehiscence or septic complications in several studies (48, 50). A weight loss of 10% or more over 3–6 months prior to surgery is considered significant.

Evidence that pre-operative nutritional support mitigates this increase in morbidity in weight-losing patients is lacking in CD but exists in other settings. The evidence supporting ~1 week of pre-operative parenteral nutrition in this setting is the strongest (92), but during recent decades the oral or enteral route has been preferred. A recent randomized trial of oral nutritional supplementation in weight-losing patients undergoing colorectal cancer resection demonstrated weight gain and fewer post-operative infectious complications in treated patients (93).

The end-point of pre-operative nutritional support is not established. It is rarely feasible to aim to regain premorbid weight. Instead, the authors consider nutritional support to be successful when weight loss has been halted so that a stable or increasing weight has been achieved (Figure 2).

### Use of Serum Albumin Concentrations in Surgery for Crohn's Disease

It is important to remember that contrary to widespread belief, serum albumin has no correlation to the nutritional state. It is well-established that undernutrition, in itself, does not affect serum albumin concentrations (94). Albumin, like other serum carrier proteins, is nevertheless important in CD, being marker of an inflammatory burden large enough to induce a degree of catabolism. Hypoalbuminaemia typically signals significant mucosal inflammation, penetrating disease with extraluminal infection, or both, and should stimulate efforts to treat those factors prior to surgery. Not surprisingly, significantly reduced serum albumin concentration (~32 g/L or less) at the time of surgery has been associated with anastomotic healing complications in several studies (49, 65), and should be taken into account when considering primary anastomosis vs. split stoma.

### Universal Enhanced-Recovery Principles

The pre-operative optimization measures discussed here apply specifically to persons undergoing surgery for DC. They should be seen as complementary to good perioperative clinical practice for colorectal surgery, based on enhanced-recovery principles (76). Such practice includes pre-operative correction of anemia, smoking, and alcohol overconsumption.

### Published Evidence on Optimization Pathways

Comprehensive and systematic pre-operative optimization thus includes measures that not only reduce perioperative risks, but it is reasonable to suggest that optimization also increases the proportion of patients where an anastomosis is safe, and where minimally invasive surgery is feasible.

Although individual components are supported by good evidence. little data is available regarding the efficacy of optimization pathways as a whole. A 2010 report from Lille, France, may be the first such study (95). Seventy-eight patients with penetrating disease underwent ileocaecal resection. Pre-operative parenteral nutrition was used in 50, EEN in 5, and percutaneous drainage of an abscess in 6. Antibiotic therapy was used in all. Thirty-five were on corticosteroid and/or anti-TNF therapy, which was weaned. Impressively, a primary anastomosis was fashioned in 72/78 patients. One patient required post-operative percutaneous drainage and three were reoperated. Following this report, the concept has been discussed in several reviews (87, 96), but there is room for further evaluation of efficacy.

## Surgery For Penetrating Crohn'S Disease

Surgery for penetrating CD is associated with higher morbidity than non-penetrating disease (97). The surgery may be technically challenging and require careful decision making (68).

The key principles of surgery for CDare:

- Avoiding intestinal anastomosis in high risk situations (sepsis, malnutrition, steroids)
- Preserving intestinal length (minimal resections and strictureplasty).

In penetrating disease, the first principle obviously applies. The principle of preserving bowel length is important although the option of strictureplasty is generally not available in penetrating/fistulating disease. Surgery for penetrating disease usually involves resection of the diseased segment with minimal margins of macroscopically normal bowel. If a very extensive resection is required which would inevitably result in short bowel syndrome then consideration of alternative approaches such as defunctioning or re-visiting medical therapy should be considered. The precise timing of surgery depends on a balance between the patient's symptoms, the natural history of the disease process, the amount of optimization required, and the details of the surgery proposed.

Most surgeries for CD may be undertaken using minimally invasive techniques. Kristo et al. report that a minimally invasive approach for penetrating CD is not associated with increased complications (98), although in some challenging scenarios a traditional open approach may be required. Any surgical approach for penetrating disease requires careful entry into the peritoneal cavity. An open Hassan technique away from previous scars, typically in the left upper quadrant may be sensible. If dense adhesions are anticipated and an open approach is used, entry into the abdomen above or below previous incisions will reduce the risk of inadvertent enterotomy.

The general principle of most operations for penetrating Crohn's is to free relevant adhesions to permit a thorough assessment of the pathology. It is important to confirm that the pathology encountered corresponds to that which was anticipated from pre-operative imaging. Any additional or unexpected findings may require a change in operative strategy.

There are several clinical situations in which surgery may be undertaken for penetrating CD (Table 2), which will be discussed in turn.

**Table 2:** Potential indications for surgery due to penetrating Crohn's disease.

Free perforation
Inflammatory mass
Abscess
Fistula
Post-operative entero-cutaneous fistula

### Free Perforation

Intestinal perforation due to Crohn's with generalized peritonitis is rare but well-recognized. Perforation most commonly involves small bowel perforation. Management involves resuscitation and prompt surgery. Surgery typically involves resection of the perforated and disease segment with the formation of a stoma. Simple repair of the perforation is destined for failure.

### Inflammatory Mass

Inflammatory disease is usually managed medically initially. Such cases must be discussed at an IBD MDT (99). Surgery may be indicated for an inflammatory mass because of persisting symptoms despite optimum medical therapy or suspicion of fistulation/sepsis driving failure of medical therapy. If a large inflammatory mass with densely adherent adjacent loops of the bowel is encountered, then the key surgical principle is to “do no harm.” If the inflammatory mass can be easily separated from adjacent non-disease structures then resection is probably appropriate. If it is impossible to differentiate between diseased and non-disease bowel, then there may be a role for a diverting stoma only, even if this results in a high output proximal stoma. Although there is no trial data to support defunctioning alone, in CD this commonly results in a decrease in the inflammatory process, which may permit more definitive and less radical surgery in a few months time.

### Intra-Abdominal Abscess

Abscesses associated with CD should usually be managed with antibiotics and radiologically guided drainage. A significant proportion of patients will avoid surgery in the acute situation and respond to subsequent medical management typically with anti-TNF drugs. Surgery is indicated if sepsis cannot be controlled non-surgically, which may be due to associated stricturing or fistulation. Antibiotics/drainage may also be used as a bridge to surgery, such that surgery can be undertaken in favorable and non-septic conditions, which may permit resection and anastomosis rather than stoma formation (100).

Surgery for Crohn's associated with an abscess will generally involve separating the affected segment of the bowel from adjacent loops or structures and ensuring that there is not an associated fistula. Checking for a fistula to an adjacent segment of the bowel or other structure may be difficult. We commonly use CO_2_ insufflation to do a “bicycle-tyre” test technique. CO_2_ is easily available from the laparoscopic stacks in theaters. The tubing may be connected to a Foley catheter which is inserted into the relevant segment of the bowel. When the CO_2_ is insufflated, usually on “low flow,” a leak from a potential fistula site will be easily apparent if present. This technique may be used to check for potential sigmoid fistulas (CO_2_/catheter inserted per rectum, duodenal fistulas (CO_2_ inserted via NG tube), or small bowel fistulas (CO_2_/catheter inserted into the open end of resected bowel). The advantage of the technique is that the CO_2_ gas gets reabsorbed within 2–3 min of insufflation. A fistula to the bladder can be checked by distending the bladder with fluid via a urethral catheter.

Usually, the diseased segment will require resection. The decision to anastomose or resect depends on factors considered as given in Table 3. Good pre-operative preparation and optimization will obviously make primary anastomosis safer.

**Table 3:** Factors to consider prior to intestinal anastomosis in Crohn's disease.

Ensure no distal obstruction of the anastomosis	Check pre-op colonoscopy and imaging
	Consider on-table colonoscopy/enteroscopy
Consider risk factors for anastomotic leakage	Sepsis
	Malnutrition
	Steroids
	Smoking
General condition of patient	Medical co-morbidity
	Haemodynamic stability intra-operatively

### Fistula

The surgical treatment of fistulating disease usually involved adhesiolysis and clarification of the precise pathology and “anatomy” of the fistulating disease. It is useful to consider which segment of the bowel is the primary diseased segment (the “donor”) and which structure is “the recipient” of the fistula. In general, the donor segment of the bowel is resected and the defect in the recipient is repaired. The decision to anastomose or to make a stoma is made after the consideration discussed above (Table 2).

Some common types of fistula warrant individual consideration:

Entero-sigmoid. A fistula between the distal ileum and sigmoid is quite common (101). The ileum is usually the donor site but occasionally significant disease can occur in both segments. It is important to have checked the sigmoid by pre-operative colonoscopy to exclude Crohn's inflammation in the sigmoid. If the sigmoid is confirmed as the recipient then ileal resection with the repair of the sigmoid defect is reasonable. Confirmation of a satisfactory sigmoid repair may be done by insufflating CO_2_ into the sigmoid via a catheter in the rectum. If the sigmoid is primarily involved or badly scarred by the fistulation process then occasionally a sigmoid resection is also required.

Enter-duodenal. Fistulation to the junction of D2/D3 is usually associated with a distal ileal disease or may occur from a previous ileo-colic anastomosis. Fistulation to D4/Djflexure is more likely to be related to the jejunal or colonic disease. The duodenum is usually the recipient segment, but careful pre-operative imaging and endoscopy are important to rule out our primary duodenal inflammation. The usual principle is to resect the donor's small bowel or colonic segment and repair the duodenum. Sometimes if the ileum can almost be “pinched off” the D2/D3 segment of the duodenum, it is convenient to fire a TA30 type stapler across the duodenum and oversew the staple line. Repairing larger defects of the duodenum usually requires thorough mobilization of the duodenum to permit tension-free transverse closure.

Entero-entero fistulae. It is quite common to find fistulae between nearby loops of the small bowel involved in a fistulating/inflammatory mass. Provided the involved loops are close to one another, it may be reasonable to resect both involved segments en bloc, provided this does not result in potential short bowel problems or unnecessarily sacrifice normal bowel. If the is an entero-enteric fistula between an inflamed terminal ileal segment of the bowel and very proximal jejunum, the principle of resecting the donor segment and repairing the recipient segment applies.

Entero-vesical or entero-vaginal. Crohn's fistulae from small bowel or colon to bladder or vagina are usually very symptomatic and require surgery (102). Surgery usually involves resection of the primarily involved bowel segment by repairing the defect in the bladder or vagina. The management of the fistulating anal disease is not discussed here.

### Post-operative Entero-Cutaneous Fistula

Post-operative sepsis and entero-cutaneous fistula may occur after surgery for CDdue to leakage from the anastomosis. Avoiding high risk anastomoses with formation and use of a 2-stage strategy will reduce the risk of ECF and associated devastating complications such as long term intestinal failure (34, 57).

### Post-operative Management

Patients should receive standard post-operative care including contemporary principles of enhanced recovery (103). A decision in the early post-operative period regarding continued Crohn's medications should be made in conjunction with the gastroenterology team. For many patients, the “default” plan should be colonoscopy at 6–12 months post-op to make a decision about subsequent drug treatment (104). Penetrating disease is probably a risk factor for recurrent disease, along with young age, and early recurrence after previous surgery. Patients at high risk of recurrence may benefit from early post-operative biologic treatment.

## Conclusion

In patients with the abdominal penetrating disease, every step is of importance with a need for meticulous planning with the joint knowledge of gastroenterologists and colorectal surgeons at the MDT. There should be an evaluation of the patient's nutritional and inflammatory status including endoscopic and radiologic evaluation of the intestines, e.g., to rule out distal obstructions. The abdominal cavity should be evaluated regarding any septic intra-abdominal complications and any visible collections should be drained and the infections should be treated with antibiotics. Meanwhile, steroids should be tapered together with optimization of the nutritional status with enteral nutrition, or parenteral if needed. At the time of surgery, experience is key. It is always important to bear in mind the use of two-staged surgery if not all parts are deemed optimal prior to or during surgery. It is after all the patient who has to carry the burden, and risks, of an anastomotic leak.

## Author Contributions

All authors listed have made a substantial, direct, and intellectual contribution to the work and approved it for publication.

## Conflict of Interest

The authors declare that the research was conducted in the absence of any commercial or financial relationships that could be construed as a potential conflict of interest.

## Publisher's Note

All claims expressed in this article are solely those of the authors and do not necessarily represent those of their affiliated organizations, or those of the publisher, the editors and the reviewers. Any product that may be evaluated in this article, or claim that may be made by its manufacturer, is not guaranteed or endorsed by the publisher.

## Abbreviations

CD: Crohn's disease
IBD: inflammatory bowel disease
ECF: enterocutaneous fistula
IF: intestinal failure
MDT: multidisciplinary team conference
EEN: exclusive enteral nutrition
MUST: Malnutrition Universal Screening Tool
IASC: intra-abdominal septic complications.
